# Acceptability of neural stem cell therapy for cerebral palsy: survey of the Australian cerebral palsy community

**DOI:** 10.1186/s13287-023-03246-2

**Published:** 2023-02-03

**Authors:** Madeleine J. Smith, Megan Finch-Edmondson, Suzanne L. Miller, Annabel Webb, Michael C. Fahey, Graham Jenkin, Madison Claire Badawy Paton, Courtney A. McDonald

**Affiliations:** 1 The Ritchie Centre, Hudson Institute of Medical Research, Clayton, VIC Australia; 2 Department of Obstetrics and Gynaecology, Monash University, Clayton, VIC Australia; 3 Cerebral Palsy Alliance Research Institute, Speciality of Child and Adolescent Health, Sydney Medical School, Faculty of Medicine and Health, The University of Sydney, Sydney, NSW Australia; 4 Department of Paediatrics, Monash University, Clayton, VIC Australia

**Keywords:** Cell therapy, Cerebral palsy, Brain injury, Stakeholder engagement, Pediatric neurology, Neurosurgery

## Abstract

**Background:**

Neural stem cells (NSCs) have the potential to engraft and replace damaged brain tissue, repairing the damaged neonatal brain that causes cerebral palsy (CP). There are procedures that could increase engraftment of NSCs and may be critical for efficacy, but hold notable risks. Before clinical trials progress, it is important to engage with the CP community to understand their opinions. The aim of this study was to determine the acceptability of NSC therapy for CP in the CP community.

**Methods:**

Australian residents with CP and parents/carers of those with CP completed a questionnaire to determine their willingness to use NSCs from three sources (fetal, embryonic and induced pluripotent stem cells) and their willingness to undergo accompanying procedures (neurosurgery, immunosuppression) that carry potential risks. To further explore their views, participants also answered free text questions about their ethical concerns regarding the source of NSCs and their perceptions of meaningful outcomes following NSC treatment.

**Results:**

In total, 232 responses were analyzed. Participants were willing to use NSCs from all three cell sources and were willing to undergo NSC therapy despite the need for neurosurgery and immunosuppression. Participants identified a range of outcome domains considered important following NSC treatment including gross motor function, quality of life, independence and cognitive function.

**Conclusions:**

Hypothetical NSC therapy was acceptable to the Australian CP community. This study has identified important findings from the CP community which can be used to inform future NSC research, including the design of clinical trials which may help to increase recruitment, compliance and participant satisfaction.

**Supplementary Information:**

The online version contains supplementary material available at 10.1186/s13287-023-03246-2.

## Background

Cerebral palsy (CP) is the most common disability of childhood and, unfortunately, there are limited effective interventions and no neuroregenerative treatments. While there are various aetiologies underlying CP, the condition is characterized by injury to the developing brain. Brain imaging has found that abnormalities and/or volume loss in white matter, gray matter and focal lesion sites are present in 50% to 76% of people with CP [1]. Stem cell therapies, including neural stem cells (NSCs), are under investigation for the prevention or repair of brain injury, and offer a scientifically plausible treatment for CP [2]. Unlike other stem cell types, including mesenchymal stem cells, umbilical cord blood cells and haematopoietic stem cells, NSCs have the ability to substantially engraft into the damaged brain [3]. Within the brain, transplanted NSCs can form the three main brain cell types: oligodendrocytes, astrocytes and neurons [4]. NSCs therefore provide neuro-regenerative promise for people living with CP, with strong, accumulating preclinical evidence for treatment of perinatal brain injury [5].

To date, we have seen a number of published clinical trials of NSCs demonstrating safety and some early efficacy [6–8], but there are important practical and ethical consideration that need to be addressed to aid further clinical translation. Firstly, NSCs were originally obtained from fetal tissue, and can now be sourced from embryonic and induced pluripotent (iPSC) stem cells [9]. NSC source may need to be considered for future translation of this therapy, since fetal and embryonic stem cells have classically been deemed ‘ethically contentious,’ although more recently public support for their use has been reported [10, 11]. Despite the availability of public opinion in the literature, the specific views of the CP community regarding the clinical use of these cell types are not known. In addition, accompanying procedures are likely to be required to increase the efficacy of NSC therapy, such as neurosurgery and immunosuppression [12, 13]. NSCs are not immunoprivileged and donor NSCs may likely be rejected by the immune system, therefore long-term immunosuppression may be required. Moreover, transplanted NSCs have limited ability to cross the blood brain barrier or choroid plexus and migrate through the damaged brain [14]. Since neuro-regeneration is the aim of this therapy, most pre-clinical studies have utilized direct injection of NSCs into the brain [5], therefore neurosurgery is likely necessary. Both accompanying procedures carry risks that may make this therapy less acceptable to the CP community. Side effects of immunosuppression may include hypertension, nephrotoxicity and increased risk of infection [15]. Additionally, neurosurgery holds separate risks such as bleeding and the risk of anesthetic and sedative use [16]. It is therefore important to gauge the opinions of the CP community given these notable considerations.

People with CP and their parents/carers are key stakeholders in the development of new stem cell treatments. It is widely recognized in regulatory bodies around the world that people with lived experience should be involved in research as early as possible [17–19]. The World Health Organization and United Nations Children’s Fund (UNICEF) Declaration of Alma-Ata states: *The people have the right and duty to participate individually and collectively in the planning and implementation of their health care* [20]. Stem cell research is well supported by the CP community [21] and many people in the CP community are already interested and optimistically engaged in the field, reflected in the growing number of people seeking stem cell therapies overseas [22]. Nevertheless, given the ethical and practical considerations associated in particular with NSC therapy for CP, it is imperative that the views of the CP community are considered alongside scientific data, to help to inform future NSC research. Thus, we aimed to determine whether people in the CP community would be willing to use NSC therapy, given considerations around cell source, neurosurgery and immunosuppression. We also determined the likelihood that people in the CP community would elect to be involved in an NSC clinical trial for CP and outcomes they deem meaningful following treatment.

## Methods

An online questionnaire titled ‘*Your Opinion: Neural Stem Cell Therapy for Cerebral Palsy*’ was distributed via Research Electronic Data Capture (REDCap) between October 2020 and May 2021 (ethics approval, University of Sydney Human Research Ethics Committee, project number: 2020/495).

### Questionnaire development

Prior to public launch, the questionnaire was piloted to optimize questionnaire length, as well as to receive feedback regarding language and respondent comprehension. Readability and comprehension feedback to optimize the types of material presented to participants was provided by a parent of a child with CP. Study concept and design was also presented to the Cerebral Palsy Alliance Stem Cell Reference Group comprising people with CP and their parents/carers to elicit additional feedback. Hudson Institute of Medical researchers with varying stem cell/research literacy to determine the time required to complete the questionnaire.

### Participants

Upon study launch, Australian residents who identified as someone with CP, or parents or carers of those with CP were invited to participate. Recruitment involved email invitations via the Australian Capital Territory and New South Wales Cerebral Palsy Registers, the Victorian Cerebral Palsy Register and the Queensland Cerebral Palsy Register. The survey was also advertised on social media and dedicated webpages via the Cerebral Palsy Alliance Research Institute and the University of Sydney. Participants were provided with study information including a warning for potentially distressing content. They then self-assessed eligibility (including that they were over 18 years old and did not have an intellectual impairment), and consented to participate in the survey.

### Questionnaire content

Lay information about stem cells was provided at the beginning of the questionnaire, followed by more detail about NSCs including relevant peer-reviewed scientific information. The REDCap questionnaire (see Additional file 1 for full questionnaire) comprised a maximum of 40 questions (depending on branching logic), including ten-point linear numeric scales, free-text responses, and ‘yes’ or ‘no’ questions. For the linear numeric scales, a response of 0–4 and 6–10 represented varying degrees of ‘negative’ or ‘positive’ support, acceptability, willingness or likelihood, respectively. A response of 5 corresponded to ‘unsure.’ Participant demographics included postcode, gender, age, identity as a person with CP or a parent/carer of someone with CP, age of their child/the person they care for, and Gross Motor Classification System (GMFCS) level to measure CP severity. Questions relating to key themes of NSC source, neurosurgery, immunosuppression, willingness to participate in clinical trials and meaningful motor improvements were included in this manuscript for analysis.

### Data analysis and statistics

Descriptive statistics were used to investigate sample characteristics. The Wilcoxon Signed-Rank test (two related groups) and Freidman’s test with Wilcoxon-Signed rank post hoc (more than two related groups) were used to assess differences across numeric scaled responses. Logistic regression was conducted to analyze differences in yes/no responses. The effects of participant demographics were analyzed using Kruskal-Wallis and Mann-Whitney U post hoc pairwise comparison (numeric scaled responses) or a logistic regression model (yes/no responses). A *p*-value of < 0.05 was considered statistically significant across analyses, except when Bonferroni adjustment for post hoc analysis was required (adjusted significance = 0.05/number of pairwise comparisons). Statistical analysis was conducted using SPSS V 26. Free-text responses were analyed using conventional content analysis [23] and were coded in an inductive manner without pre-determining categories. Two authors (MS and MP) reviewed all participant free text responses and coded 25% of these independently. The authors then met to discuss consistency of code selection. Both MS and MP then coded the entire dataset using these agreed codes and any discrepancies were resolved through discussion. MS and MP developed categories and sub-categories.

## Results

A total of 329 people consented to participate in the survey. Of these, 97 were excluded due to the following reasons: non-Australian resident (*n* = 17); self-identified as having an intellectual impairment (*n* = 1); did not progress past the demographics section of the questionnaire (*n* = 69); or duplicate responses (*n* = 10). Consequently, 232 responses were included in the analysis. Table 1 presents demographics of included survey participants. The highest proportion of participants were female (83.6%, *n* = 194) and aged between 40 and 49 (33.2%, *n* = 77). Participants with CP were mainly under the age of 29 (40%, *n* = 10) and had a GMFCS level of II (44.0%, *n* = 11). Most parents or carers of someone with CP indicated that their child/person they care for was under the age of 29 (93.2%, *n* = 193) and demonstrated a range of CP severities (GMFCS). Nearly half of the participants were from Victoria (47.3%, *n* = 98), followed by New South Wales (25.6%, *n* = 53) and Queensland (17.9%, *n* = 37). Distribution of participants across these three states is generally reflective of population distributions of people with CP as reported in the Australian CP Register Report 2018 [24]. Notably, more than two-thirds of survey respondents lived in a high socio-economic area (SEIFA 6–10, 68.4%, *n* = 141).

**Table 1:** Participant demographics (*n* = 232)

Demographic characteristics	Person with CP (*n* = 25, 10.8%) *n* (%)	Parent/carer of someone with CP (*n* = 207, 89.2%) *n* (%)	Total *n* (%)
*Gender*			
Male	8 (32.0)	29 (14.0)	37 (15.9)
Female	16 (64.0)	178 (86.0)	194 (83.6)
Other	1 (4.0)	0	1 (0.4)
*Age of participant*			
18–29	10 (40.0)	13 (6.3)	23 (9.9)
30–39	6 (24.0)	58 (28.0)	64 (27.6)
40–49	4 (16.0)	73 (35.3)	77 (33.2)
50–59	5 (20.0)	45 (21.7)	50 (21.6)
60 and over	0	18 (8.7)	18 (7.8)
*Age of person with CP (parent/carer answering)*			
Under 29	-	193 (93.2)	-
30–39	-	10 (4.8)	-
40–49	-	3 (1.4)	-
50–59	-	1 (0.5)	-
60 and over	-	0	-
SEIFA*			
Low (1–5)	9 (36.0)	65 (31.6)	74 (32.0)
High (6–10)	16 (64.0)	141 (68.4)	157 (68.0)
*State*			
ACT	1 (4.0)	6 (2.9)	7 (3.0)
NSW	9 (36.0)	53 (25.6)	62 (26.7)
QLD	7 (28.0)	37 (17.9)	44 (19.0)
SA	2 (8.0)	5 (2.4)	7 (3.0)
TAS	1 (4.0)	4 (1.9)	5 (2.2)
VIC	5 (20.0)	98 (47.3)	103 (44.4)
WA	0	4 (1.9)	4 (1.7)
*GMFCS level of person/child with CP*			
Level I	6 (24.0)	33 (15.9)	39 (16.8)
Level II	11 (44.0)	49 (23.7)	60 (25.9)
Level III	3 (12.0)	28 (13.5)	31 (13.4)
Level IV	3 (12.0)	39 (16.8)	42 (18.1)
Level V	0	50 (24.2)	50 (21.6)
Unknown	2 (8.0)	8 (3.9)	10 (4.3)

^*^SEIFA was derived from postcode data using the Australian Bureau of Statistics product 2033.0.55.001—Census of Population and Housing: Socio-Economic Indexes for Areas (SEIFA), Australia, 2016

### Very high acceptability of stem cell research

At the beginning of the questionnaire 88.7% (*n* = 206) of participants were highly accepting of stem cell research *in general* (defined as a score of 7–10/10). Additionally, 86.2% (*n* = 200) of participants were highly accepting of stem cell research *for CP*. GMFCS level of the child/person cared for significantly influenced both the acceptability of stem cell research *in general* and *for CP* (*p* = 0.050 and *p* = 0.014), but post hoc analysis was underpowered to detect differences between specific groups (GMFCS levels).

### High willingness to use NSCs from various sources to treat CP

**Fig. 1:**
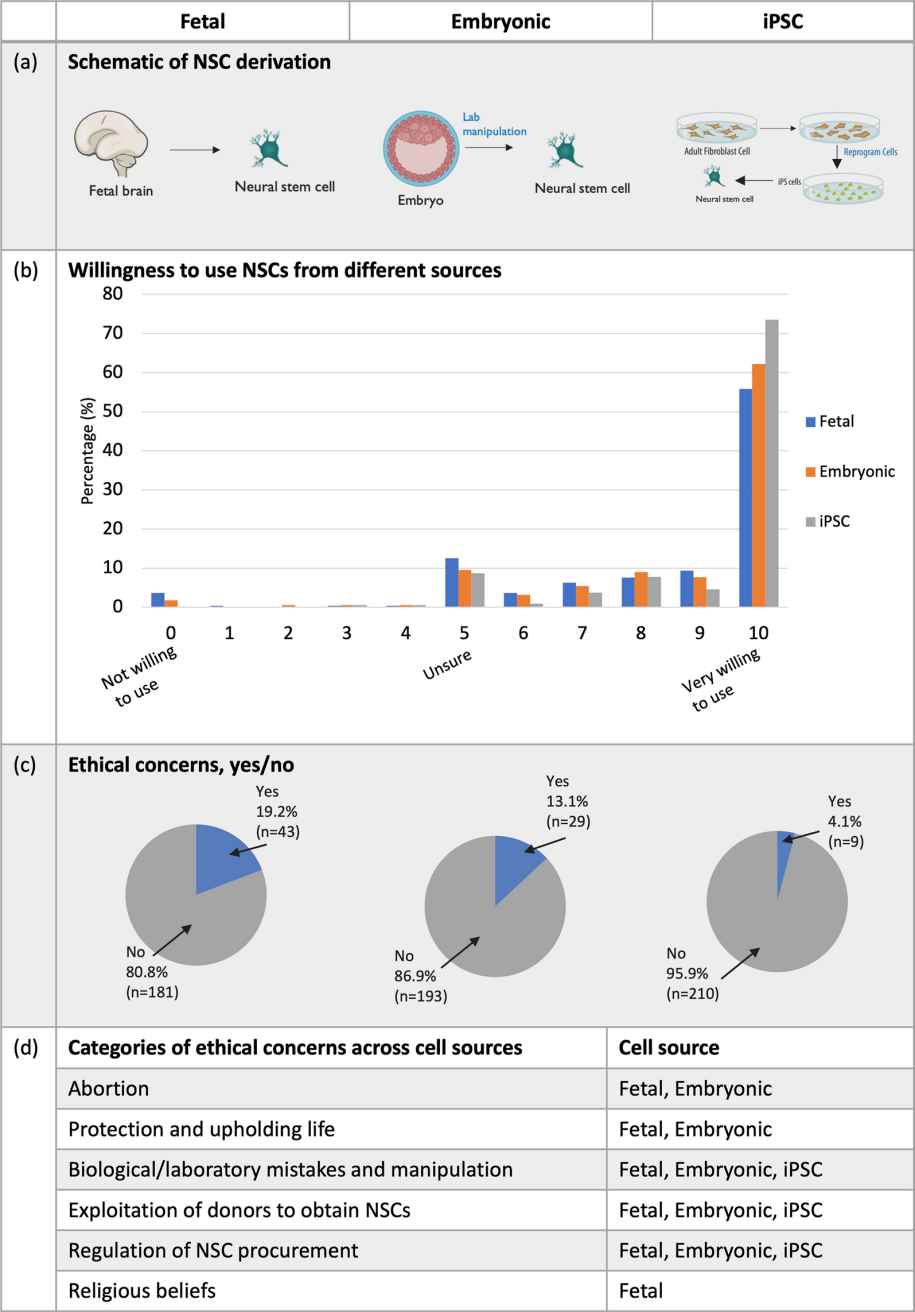
Willingness to use three neural stem cell (NSC) sources. **a** Sources of NSCs were presented to participants with the aid of schematic diagrams, created using images sourced from Biorender.com. Participants were asked about **b** their willingness to use these three NSC sources (fetal, *n* = 224; embryonic, *n* = 222; induced pluripotent stem cell (iPSC), *n* = 219) to treat CP in themselves or their child/person they care for and **c** whether they have ethical concerns about these cell sources. If they responded with ‘yes’ to ethical concerns, they were prompted to list their ethical concerns. Ethical concerns were then analyzed using content analysis, with categories of ethical concerns shown **d**. Categories of ethical concerns were raised for different combinations of cell types, indicated by the right column titled ‘cell source.’

The majority of participants were willing (defined as a score of 7–10/10) to use all three types of NSCs (Fig. 1a) as a treatment for either their own CP or for their child/person they care for with CP (fetal, 79.1%; embryonic, 84.3%; iPSC, 89.6%) (Fig. 1b). Comparing the willingness to use NSCs across the three cell sources, participants were significantly more willing to use embryonic-NSCs compared to fetal-NSCs (*p* = 0.004) and more willing to use iPSC-NSCs compared to both fetal- and embryonic-NSCs (*p* = 0.000 and *p* = 0.001, respectively). GMFCS level of the child/person being cared for was associated with a significant difference in the willingness to use iPSC-derived NSCs (*p* = 0.036). However, post hoc analysis was underpowered to detect differences between specific groups. Additionally, participant age was associated with a significant difference in the willingness to use embryonically-derived NSCs (*p* = 0.028) with post hoc analysis showing that participants aged 30–39 were more willing to use embryonically-derived NSC than participants over 60 (*p* = 0.005).

### Low ethical concerns for all three NSC sources

The majority of participants indicated that they had no ethical concerns about NSCs sourced from fetal, embryonic or iPSC origins (80.8%, 86.9%, 95.9%, respectively) (Fig. 1c). For those who indicated they did have ethical concerns, significantly more participants had ethical concerns relating to fetal-NSCs (*p* < 0.001) and embryonic-NSCs (*p* = 0.001), compared to iPSC-NSCs. To explore the detail of the ethical concerns for each cell source, if a participant selected ‘yes’ they were prompted to list their ethical concerns via free text fields. Content analysis of these text responses generated six categories (Fig. 1d) including abortion, protection and upholding of life, biological/laboratory mistakes and manipulation, exploitation of donors, regulation of NSC procurement and religious beliefs, with some variation in the categories arising for different cell sources. These six categories contained 11 subcategories and are detailed in Table 2.

**Table 2:** Qualitative analysis of ethical concerns related to three sources of neural stem cells

**Category**/*subcategories*	**Description**	**Example response/s**
**Abortion**	Anti-abortion, concerns about abortion	“(I am) anti-abortion”
*Generation of an abortion industry*	Generation of an abortion industry and commercialisation	“If it's paid and generates a market for abortions.”
*Monetary incentivisation of abortion*	Donors being swayed or incentivised to undergo an abortion by compensation or payments	“Financial remuneration could end up being a part of the process and therefore sway decision making for those considering elective abortion.”
*Abortion conflicts with personal/moral beliefs*	Personal, ethical and moral views about abortion shaping the acceptability of stem cell source	“Although I think abortion should be legal and safe, I'm not sure that it's an ethical choice…” “Is it morally right to use cells from an aborted fetus?”
**Protection and upholding of life**	The need to protect and uphold life of the fetus/embryo, including participants that stated they were pro-life	“I believe it is a human deserving of life.”
*Developmental status of the embryo or fetus*	Fetuses or embryos used at late developmental stages and personal beliefs around what constitutes “life.”	“Need to have upper limit of age of fetus that can be used—blastocyst is fine in my view.” “At what point is a fetus brain a baby brain?”
*Fetus or embryo has equivalent value to any human life*	Fetuses have the same status as a human being. Additionally, NSCs are living and should be treated as such	“Would not want to take away from someone’s life.”
**Biological/laboratory mistakes and manipulation**	Medical/laboratory manipulation of fetuses, embryos and NSCs including mistakes, errors and health of the therapeutic product	“Any laboratory manipulation must not result in errors in coding or mutation.” “Must be obtained from a healthy IVF embryo.”
**Exploitation of donors**	Unfair treatment of people donating NSCs	“Really exploitation is my main concern of vulnerable populations.”
*Exploitation of mothers/parents*	Exploitation, coercion and persuasion of donors as well as concerns around vulnerability of people donating NSCs	“Some concerns around persuasion of mothers…” “Exploitation of women in a time of uncertainty.”
Exploitation of adults to obtain donor tissue*	Exploitation, coercion and risk of harm for vulnerable adults including illegal organ harvesting, e.g., medical patients and prisoners	“There is a lot of 'medical research' (often involves harvesting of organs) being done on prisoners in China; & I don't like that idea at all.”
*Inadequate informed consent*	Adequacy/clarity of consent processes and extent of voluntary donation	“That they are willingly donated and not taken unethically or without knowledge.” “Ensuring consent for donation is clear.”
*Monetary incentivisation to donate a potential therapeutic product*	Risk of incentivising donation with renumeration of donors	“If women are paid to undergo IVF to generate available embryos.”
**Regulation of NSC procurement**	How NSCs are sourced and obtained, including the need for regulation around NSCs to ensure they are ethically sourced and the potential for commercialisation is mitigated	“That they (cells) are ethically…collected.” “Needs reasonably strict regulation.”
*Solely generating NSCs for therapeutic use*	Women may become pregnant or that embryos would be created with the sole intention of creating NSCs for therapeutic use	“The risk of parents falling pregnant specifically to gain the cells of their fetus doesn't sit well with me.” “Would not want fetuses to be produced for the purpose of research.”
**Religious beliefs^**	Religious beliefs informing ethical concerns and acceptability of NSC sources	“(This) conflicts with my religious beliefs.”

^*^Ethical concerns raised for induced pluripotent stem cell-derived neural stem cells only, ^Ethical concerned raised for fetally-derived neural stem cells only

Abbreviations: IVF—in vitro fertilization; NSC—neural stem cell

### General willingness to use neurosurgery and immunosuppression as accompanying procedures to NSC therapy, with a level of hesitancy

**Fig. 2:**
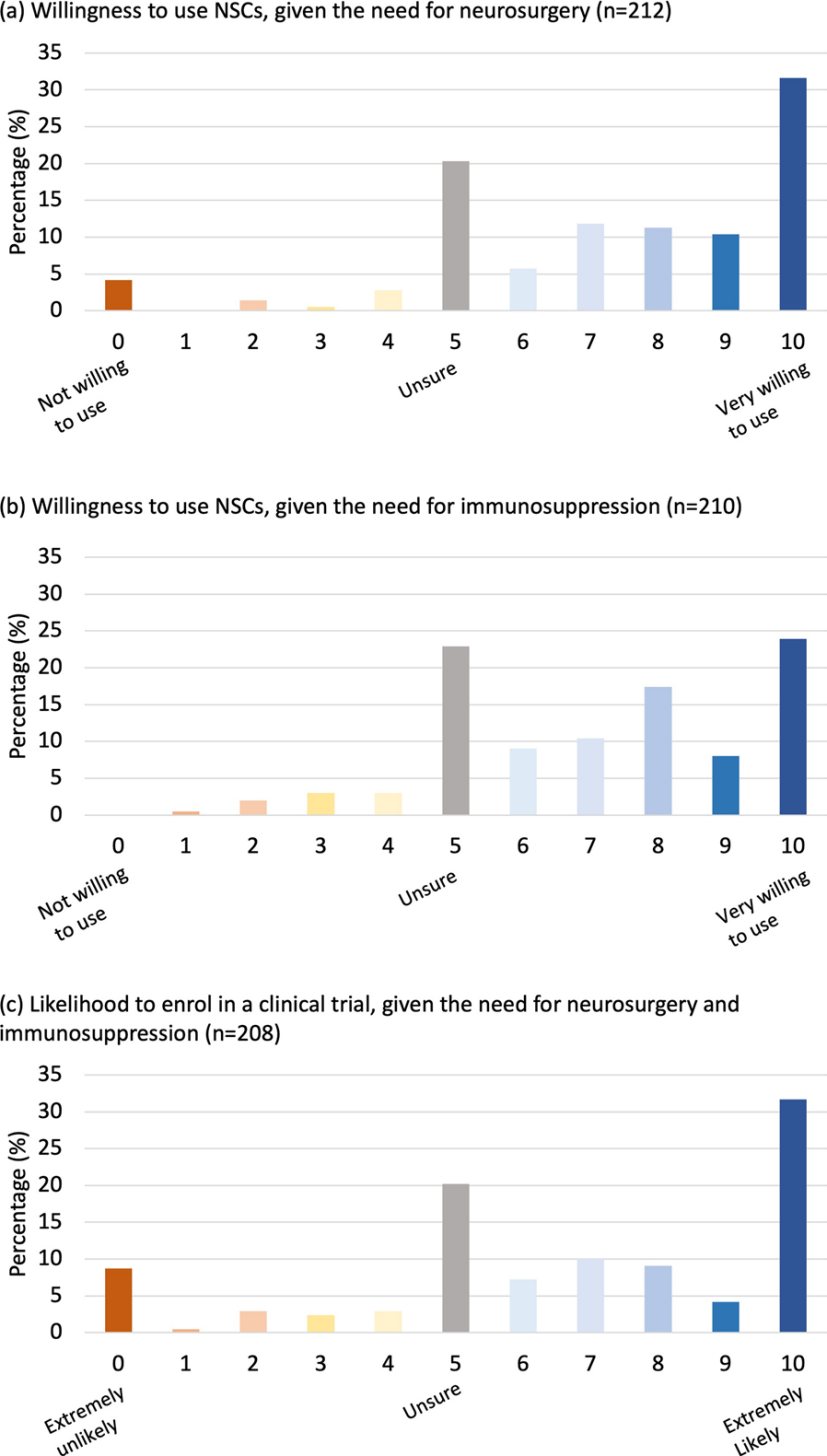
Willingness to use accompanying procedures and participate in a clinical trial. Participants willingness to use neural stem cells (NSCs) given the need to administer them **a** via neurosurgery and **b** in conjunction with immunosuppression. **c** Likelihood that they would elect to enroll in a clinical trial, given the need for neurosurgery and immunosuppression as an accompanying for NSC treatment

Neurosurgery and immunosuppression are likely to be a necessary part of an efficacious NSC therapy [12, 13]; however, both are associated with increased risks. Participants considered a list of benefits of neurosurgery including that it allows NSCs to be given directly to the site of injury, allows NSCs to be most effective and is the only method of administering NSCs which replaces dead brain cells. The risks presented to participants included 1–2% risk of bleeding, 1–2% risk of infection and < 1% risk of allergy to anesthetic. Potential risks of immunosuppression that were presented to participants included reduced immune system function, for at least 9 months. The benefits of immunosuppression presented to participants included prolonged NSC survival in the brain, increasing NSC benefits which could lead to greater cognitive and motor improvements. The majority of participants (65.1%) indicated a willingness to use NSC therapy (defined as a score of 7–10/10) given the need for neurosurgery (Fig. 2a). Additionally, 59.7% of participants were willing to use NSCs given the need to use immunosuppression (Fig. 2b). There was a notable level of uncertainty for both accompanying procedures, as the second most frequent response was ‘unsure’ for both neurosurgery and immunosuppression (20.3% and 22.9%, respectively). In addition, participants were significantly less willing to use immunosuppression compared to neurosurgery (*p* = 0.003).

Participants were likely to participate in an NSC clinical trial (55.2%), even with the need for neurosurgery and immunosuppression, with the most frequent response being ‘extremely likely’ (30.6%, *n* = 57) followed by ‘unsure’ (21.0%, *n* = 39) (Fig. 2c). Participant demographics did not significantly influence responses.

### Support for NSC therapy increases with recipient age

**Fig. 3:**
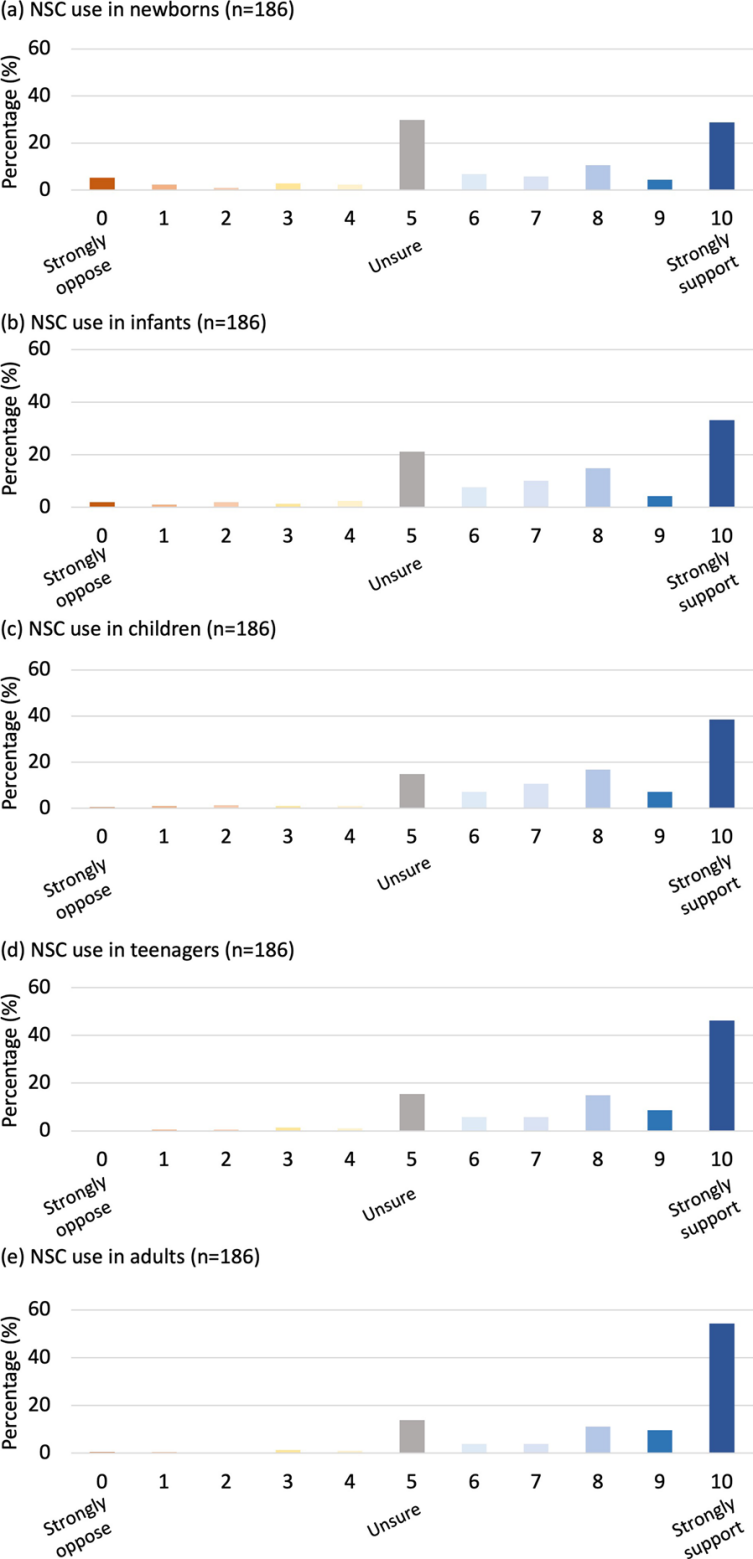
Support for NSC therapy across patient ages. Participant support of neural stem cell (NSC) therapy given the need to administer them via neurosurgery and in conjunction with immunosuppression across five age groups: **a** newborns, 0–3 months, **b** infants, 0–18 months, **c** children, 18 months and over, **d** teenagers and **e** adults

Brain injury that results in CP usually occurs during pregnancy or around the time of birth. Evidence from preclinical studies suggests that early treatment with stem cells following injury leads to the best outcomes [25]. We therefore wanted to gauge whether support for NSC therapy varied across five recipient age groups (from newborns to adults), given the need to use both neurosurgery and immunosuppression (Fig. 3). The most frequent response for newborn recipients was ‘unsure’ (30.1%, *n* = 56) followed by ‘strongly support’ (28.0%, *n* = 52). Interestingly, the most frequent response for infants, children, teenagers and adults was ‘strongly support’ (32.8%, 38.7%, 44.1%, 52.7%), and support significantly increased (*p* < 0.005, in a post hoc analysis) as the proposed recipient age increased. Participant demographics did not significantly influence responses for these questions.

### Defining a range of meaningful treatment effects across motor and non-motor outcome domains

Based on their lived experience of CP, participants were asked what would constitute a small but significant improvement in motor function, if they were to consider receiving a stem cell treatment in future. Participants were presented with a hypothetical list of considerations regarding stem cell therapy including a 15% chance of infection, 2% risk of other adverse events and a 10–20% improvement in motor function. From participants free-text responses, content analysis generated seven categories of treatment effects (and nine subcategories) including gross and fine motor function but also various other non-motor domains including activities of daily living, cognitive function and intellectual capacity, communication, independence, quality of life and “any improvement” (Table 3).

**Table 3:** Categories and subcategories of meaningful treatment effects based on lived experience with cerebral palsy

**Category**/*subcategories*	**Description**	**Example response/s**
**Motor function**	Improved ability to perform voluntary movement and control involuntary movement	“Any improvement in motor function would be wonderful for my son as he does not have any independent/intentional movement.”
*Fine motor function*	Improvement in small movements using the hands and smaller muscle groups, like the fingers	“More fine motor control in fingers/hands better able to grasp and manipulate things.”
*Gross motor function*	Improvement in skills that involve large muscle groups, e.g., standing, walking, running, crawling, stability, flexibility, balance and coordination	“…if his walking improved just a tiny bit.”
*Motor disorders of CP*	Reduction in the symptoms of motor disorders including spasticity, dystonia and ataxia	“Less spasticity in my leg muscles.”
**Activities of daily living**	Increased ability to perform essential and routine daily tasks	“Increased ability in day to day functions.”
*Self-care*	Increased ability to perform essential everyday tasks to care for oneself, e.g., grooming, eating, bathing and toileting	“For her to be able to hold a fork or spoon and feed herself.”
*Transfers*	Improved transfers, e.g., from wheelchair to commode, in and out of vehicle	“Easier transfers from wheelchair to bed or toilet and back.”
*Socialization*	Improved socialization, e.g., play, keeping up with peers, sport, crafts and music	“Play like other children.”
**Cognitive function and intellectual capacity**	Improved cognitive function, e.g., learning, understanding and intellect	“Improve her intellectual/mental capacity.”
** Communication**	Increased ability to communicate, e.g., improved speed and clarity of speech, use of communication devices	“If you could tell me it would likely help him speak, or use his hands to sign clearly, I'd probably take the risk.”
**Independence**	Increased independence for the person with cerebral palsy or parents/carers, including reduced reliance on parents/carers	“If it achieves independence or a level of independence then that would be worth the risks.” “If my brother can help himself get ready or be able to transport himself even a little bit without assistance from the family, that would be great.”
*Dependence on interventions*	Reduced use or independence from physical therapy, drugs, surgery, devices and interventions, e.g., botox, ankle–foot orthosis	“…the ability to give up his Botox injections.” “…to reduce recurrent surgeries for hips or spine.”
**Quality of life**	Improved quality of life for person with cerebral palsy, parent, carer or family	“…improvement in their quality of life and the quality of life for the family.”
*Physical*	Reduced pain, fatigue and risk of injuries	“I'd love to be able to have better days then worse days. To wake up with no pain.”
*Psychological*	Happier life, dignity and confidence	“It would mean inclusion. And validation to her that she is a valuable asset. Currently she is spoken over, ignored, or spoken down to.”
**Any improvement**	Any improvement across any domain deemed “worthwhile”	“We would take any improvement we can get.”

## Discussion

This is the first study to survey people in the Australian CP community about the acceptability of NSC therapy to treat CP. For many years there has been extensive public debate about the use of embryonic and fetal stem cells [26], which are the primary sources of NSCs. Additionally, ethical concerns about these cell sources continue to be cited by researchers and clinicians as limitations for their therapeutic use [27]. Despite these assumptions, a number of studies over the past decade have established that both public perceptions and media discourse have shifted since the early years of stem cell research, with stem cell treatments no longer broadly generalized as ethically controversial [11, 28, 29]. While we have now seen a number of clinical studies utilizing NSC treatment for children with CP in early phase trials [6–8], public perspectives on the use of NSCs are varied and it was important to quantify CP community perceptions regarding the use of these cells. We found overall, > 75% of participants were willing to use NSCs across all three cell sources (fetal, embryonic and iPSC), supporting the use of these cell types as a potential treatment for CP. This is consistent with a recent study that showed that a subset of the Australian population supports the use of embryonic and fetally-derived cells for the treatment of neurological conditions [11]. Some ethical concerns identified related to appropriate regulation, product quality and ensuring donors are not exploited. These concerns can be addressed by implementing legislation and guidelines around the use of NSCs. Similar sentiments have been reported in a study of Canadian adults which showed participants were accepting of human embryonic stem cells, if there is strict regulation [30]. Additionally, a small proportion of our participants had ethical concerns related to religion, abortion and the protection and upholding of life, which reflect traditional ethical reasons against using these cell sources [31]. It is important not to dismiss individuals that may have concerns about the use of these stem cells; however, these should be contextualized with the current findings of this study from our relevant population that shows the majority did not have ethical concerns.

Participants were willing to use NSCs, despite the requirement to undergo ‘risky’ neurosurgical and immunosuppressive procedures and indicated a willingness to enroll in a clinical trial for NSC therapy. This is consistent with a survey of people with spinal cord injury which revealed that participants would take on a specified level of risk in stem cell clinical trials (e.g., 1% risk of infection) [32]. These results are not surprising, as we know that many patients are willing to travel to receive potentially risky stem cell treatment, at great financial cost [33, 34]. Despite overall acceptability, there was a notable level of uncertainty among survey respondents since the second most frequent response was ‘5—unsure’ for both accompanying procedures (neurosurgery and immunosuppression). Some uncertainty may be attributed to the fact that the source of NSCs was not specified for these questions, and some respondents may have answered differently depending on the cell source. Furthermore, there is limited published safety and efficacy data available to support the accompanying procedures in combination with NSCs for the treatment of CP. As such, limited scientific data were available to be provided to participants, and this could explain some of the reported hesitancy. If true, it may be possible that hesitancy around the use of immunosuppression could be overcome through further research to generate robust safety data and proven efficacy of immunosuppression with NSCs for CP, combined with education of participants. Use of education to support stakeholder decision-making is supported by a recent study of clinicians that found their likelihood to refer patients into a stem cell clinical trial using immunosuppression was low, but significantly increased following an educational workshop [35]. Although there is acceptance for these procedures, this does not change the fact that these procedures hold inherent risk and this may result in uncertainty during stakeholder decision making. Adding to this uncertainty was the lower participant acceptability of NSC therapy in younger patients. To help address this, clear safety and efficacy data across age groups, but also in general, will be required to increase patient confidence.

Traditionally, when researching treatments for CP, outcomes are measured by improvements in motor control. However, we know from anecdotal evidence that people living with CP value improvements in other domains, and there is no consensus on what constitutes a meaningful improvement in a clinical trial of a stem cell treatment for CP [27]. Our results showed that participants value a range of motor improvements, but also extended their responses, without prompt, to non-motor domains including quality of life, independence and communication. Many of our reported categories are reflective of the National Institute of Neurology Disorders Common Data Elements for CP [36], which categorized data from studies in children and young people with CP into six domains. It was developed with the aim to standardize data collection to increase efficiency and effectiveness of clinical studies. Our results further bolster the need to expand the outcome domains used in clinical trials to assess improvements that are meaningful to people with CP and their families/carers. Additionally, a number of participants said that “any improvement” would make NSC therapy worthwhile. This is an important finding since systematic review evidence indicates that stem cell treatment offers a small but significant improvement in motor function [2], aligning with our community perspectives and expectations.

We acknowledge the limitations of this study, including that information provided to participants was limited by the interplay between feasible study length, comprehension of the audience and to accommodate for the specificity of the data surrounding NSCs, neurosurgery and immunosuppression. A different format with more information provided to participants may have provided more relevant data; however, this would have reduced the capacity to achieve a large number of participants. The results from this study may need to combined with more in-depth questionnaire or in-person interview. We endeavored to provide a balance between hypothetical risks and benefits; however, in reality these statistics would be highly variable in a clinical setting. Additionally, there are several factors that limit the generalizability of these results to the wider CP community. Firstly, only people in the CP community living in Australia and who could speak English were eligible to participate and we did not collect information about participant ethnicity, thus it is likely that important opinions representing a more diverse community were not captured. Similarly, we did not collect information on participants’ religiosity and we could not determine sources of influence on the acceptability of the three cell sources. Future studies should be designed to target participants from varied countries, socioeconomic status and religious beliefs. Participation in this survey was limited by our recruitment strategy to those engaged in the CP community via Australian CP Registers or on social media. Additionally, a small sample of those with CP limited our ability to investigate whether their responses differed from parents/carers. Responses from parents/carers are important as they often act as the person responsible for providing consent of children in research, where appropriate. In our study, there was no difference statistically to indicate that those with CP and parents/carers would have different responses. However, children and adults with CP are rarely asked about preferences and future research should prioritize capturing this information in a larger sample via more inclusive methods. More inclusive methods could include focus groups and one-on-one interview style formats. Finally, international attitudes of people in high-income countries are likely to be similar, but more research is warranted to infer equivalence. Broadly, all reasons mentioned limit the generalizability of study findings to other populations of people with CP.

## Conclusion

NSCs have the potential to replace injured brain tissue in CP and reduce symptoms/improve function, but complex considerations around the therapeutic use of NSCs require stakeholder and community opinions. Here we have shown that people in the Australian CP community were supportive of NSC therapy even when ethical and practical considerations were provided. Limitations of this study include the generalizability of results due to recruitment strategy, the type and amount of information presented to participants within a short online survey, and the small sample of participants with CP compared to the number of parents/carers of those with CP. Further effort should aim to understand hesitancies in the CP community to identify what further research is needed to address those concerns by providing stronger evidence. This could be determined using other methods such as small focus groups and one-on-one interviews. Canvassing the CP community has provided important information that may help to design and plan the translation of a clinically-relevant and acceptable NSC therapy.

## Supplementary Information

**Additional file 1**. Questionnaire in full as provided to participants.

## Data Availability

De-identified datasets analyzed during the current study are available from the corresponding author on reasonable request.

## Abbreviations

CP: Cerebral palsy
GMFCS: Gross motor function classification system
NSC: Neural stem cells
iPSC: Induced pluripotent stem cell
SEIFA: Socio-economic indexes for area
