# Supplementary material for: Acceptability of neural stem cell therapy for cerebral palsy: survey of the Australian cerebral palsy community
Source: Stem Cell Res Ther. 2023 Feb 3;14:18. doi: 10.1186/s13287-023-03246-2 (PMC9898914; doi:10.1186/s13287-023-03246-2)
Supplement: Supplementary file 1 — Additional file 1. Questionnaire in full as provided to participants. [file 13287_2023_3246_MOESM1_ESM.pdf]

Additional File 1: Questionnaire as it appears in REDCap

Your opinion: Neural Stem Cell Therapy for Cerebral Palsy

What is this survey about?

Stem cells hold potential to treat cerebral palsy. We would like to understand your opinions on one particular type of stem cell, neural stem cells. Scientists are still in the early phases of research and it is extremely important to understand your opinion on this potential stem cell therapy, because there are no studies assessing cerebral palsy community perceptions.

Who is running this survey?

This survey is being carried out by Madeleine Smith, a PhD student at Monash University. This will take place under the supervision of Dr Madison Paton, Research Fellow at the Cerebral Palsy Alliance Research Institute, University of Sydney.

What will the survey involve for me?

If you choose to participate in this survey, you will be presented with scientific information including text and figures regarding neural stem cells. We will ask you to evaluate this information, followed by a series of questions about your opinion on specific aspects of the therapy. Outcomes from this survey will be used to inform future research, policy makers and clinicians, as we look towards translating neural stem cell therapy to human clinical trials.

How much of my time will the survey take?

This survey will be conducted online in English and will take approximately 25 minutes to complete. There is no time limit on this survey and you may save and return to the survey as you wish. Once an answer is complete, you cannot return to that question.

Do I have to be in the survey? Can I withdraw from the survey once I've started?

Taking part in this survey is completely voluntary. If you wish to stop the survey at any time, close your internet browser. Submitting your completed questionnaire is an indication of your consent to participate in the study. You can withdraw your response once you have completed the survey by contacting Madeleine Smith via email (madeleine.smith@monash.edu).

Are there any risks with being in this survey?

Some questions are personal, including a question about the person you care for or your cerebral palsy diagnosis (Gross Motor Function Classification System levels).

Some participants may be uncomfortable with themes included in this survey, such as the discussion of how neural stem cells are obtained from fetal sources (elective abortions) and the concept of treating cerebral palsy with neural stem cells. Some participants may find these themes distressing for a variety of reasons.

Results from animal research will be discussed.

If any themes cause distress and you wish to speak with someone please call Lifeline on 13 11 14 (Australia), National Suicide Prevention Lifeline on 1800 273 8255 (United States) or Canada Suicide Prevention Service on 1 833 456 4566 (Canada). You may also consider speaking with your General Practitioner (GP) or Family Practitioner for additional support.

Are there any benefits associated with being in the study?

This survey aims to gather your opinions about neural stem cells, which could inform our future research and could help ensure stem cell therapies are relevant to people in the cerebral palsy community. We do not expect the results of this survey to have any direct or immediate benefit to the participants.

What will happen to information about me that is collected during the study?

All information gathered in this survey is confidential and can only be accessed by Madeleine Smith and Dr Madison Paton. All data gathered will be stored securely via the cloud on the University of Sydney's version of REDCap. Study findings may be published, presented at scientific conferences and written up in Madeleine Smith's PhD thesis, but you will not be individually identifiable. You will have access to the results of this survey at the study's completion, via a report emailed to you once this study is finished.

What if I have a complaint or any concerns about this study?

Research involving humans in Australia is reviewed by an independent group of people call a Human Research Ethic Committee (HREC). The ethical aspects of this study have been approved by the HREC of the University of Sydney (protocol number: 2020/495). As part of this process, we have agreed to carry out the study according to the National Statement on Ethical Conduct in Human Research (2007). This statement has been developed to protect people who agree to take part in research studies.

If you are concerned about the way this study is being conducted or you wish to make a complaint to someone independent from this study, please contact the university using the outlined details below. Please quote the study title and protocol number.

The Manager, Ethics Administration, University of Sydney:

· Telephone: +61 2 8627 8176

· Email: human.ethics@sydney.edu.au

· Fax: +61 2 8627 8177 (Facsimile)

By clicking ‘Yes,’ I acknowledge that:

· I am over 18

· I am a permanent resident in Australia, US or Canada

· I have cerebral palsy or I am a parent/carer of someone with cerebral palsy

· I do not have a known intellectual impairment

· I am voluntarily taking part in this survey and I can withdraw at any time

· I have been given information about the nature and purpose of this study

· I can ask questions about this research survey at any time

· I understand that the results of this study may be published, and that publications will not contain my name or any identifiable information about me

· I understand that this study is being carried out in a manner conforming to the National Statement on Ethical Conduct in Human Research (2007)

· I have read and understood the information in this consent form

Your contact for this survey if you have any further questions is Madeleine Smith: madeleine.smith@monash.edu

---

1) Do you meet the above inclusion criteria and do you agree to participate in this survey?

◯ Yes
◯ No

Survey welcome

Thank you for agreeing to participate in our research survey: Your opinion: Neural Stem Cell Therapy for Cerebral Palsy

The following questions will ask for some contact details and some information about you.

Survey 1% Complete

1. Information about you

Contact Information	
First Name	________________
Last Name	________________
E-mail	________________
Do you have a known intellectual impairment?	◯ Yes<br>◯ No
What is your country of residence?	◯ Australia<br>◯ US<br>◯ Canada
Please enter your postcode/zipcode:	________________
What is your gender:	◯ Male<br>◯ Female<br>◯ Other
Age (years):	◯ Under 20<br>◯ 20-24<br>◯ 25-29<br>◯ 30-34<br>◯ 35-39<br>◯ 40-44<br>◯ 45-49<br>◯ 50-54<br>◯ 55-59<br>◯ Over 60
Please select a response that is most relevant to you:	◯ I have cerebral palsy<br>◯ I am a parent of someone with cerebral palsy<br>◯ I am a carer of someone with cerebral palsy (not the parent)
What is the age of your child/person you care for:	◯ Under 20<br>◯ 20-24<br>◯ 25-29<br>◯ 30-34<br>◯ 34-39<br>◯ 40-44<br>◯ 45-49<br>◯ 50-54<br>◯ 55-59<br>◯ Over 60

If known, what is the severity of cerebral palsy experienced by your child or the individual you care for? (reported in Gross Motor Function Classification System (GMFCS) levels)
If your child's GMFCS level varies, please select the GMFCS level your child would fall into on their worst day.
Please see image below for reference.

◯ Unknown
◯ Level I
◯ Level II
◯ Level III
◯ Level IV
◯ Level V

If known, what is the severity of your cerebral palsy? (reported in Gross Function Classification System (GMFCS) levels)
If your GMFCS level varies, please select the level that would apply to you on your worst day.
Please see image below for reference.

◯ Unknown
◯ Level I
◯ Level II
◯ Level III
◯ Level IV
◯ Level V

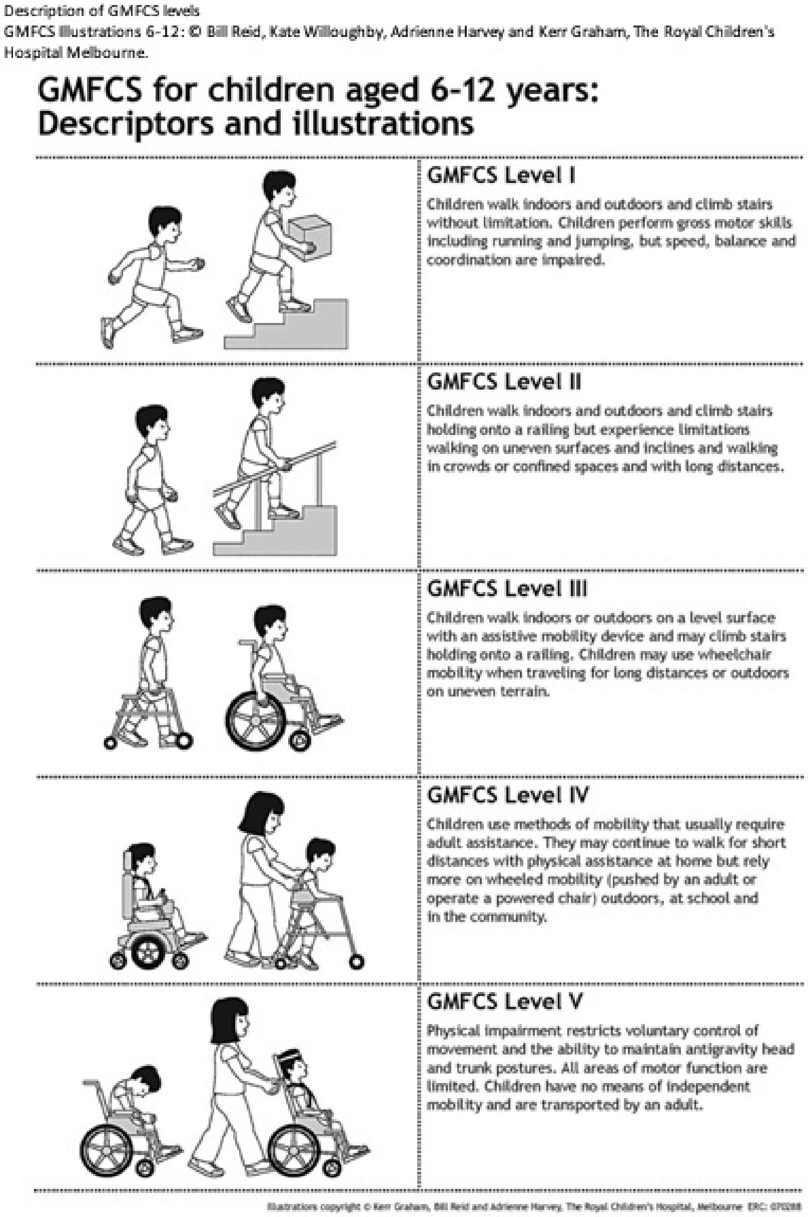

You will now be presented with some information on stem cells

Cells are the building blocks for every part of our body, which are formed from stem cells.

Stem cells are able to develop into specialised cell types in the body.

Stem cells are unique from other cells because they can:

- Develop into more specialised cell types

- Make copies of themselves

Stem cells can turn into different cells such as skin cells, muscle cells or bone cells. They can repair tissue in the body in response to injury. Additionally, scientists have been researching stem cells to treat disease, which is known as regenerative medicine.

Stem cells are obtained from different sources including:

- Adult tissue: e.g. fat tissue, bone marrow

- Fetal tissue: cells obtained from fetuses sourced from elective abortion or miscarriage, as a donation to research

- Tissues of pregnancy: e.g. placenta

- Embryos: cells obtained from embryos that are no longer needed for IVF, with consent from the individuals that have undergone IVF

- Blood: e.g. umbilical cord blood cells

In most countries stem cells are only used to treat blood cancers, for skin grafting and some autoimmune diseases. There is yet to be a proven stem cell treatment for cerebral palsy, but different stem cell types are currently being researched. There are currently more than 10 human research trials, known as clinical trials, underway internationally using stem cells to treat cerebral palsy.

---

14) We want to know your opinion prior to giving you more information about stem cells.

Based on your current knowledge, how acceptable is stem cell research?

◯ 0 - Totally unacceptable
◯ 1
◯ 2
◯ 3
◯ 4
◯ 5 - Neutral
◯ 6
◯ 7
◯ 8
◯ 9
◯ 10 - Perfectly acceptable

---

15) How acceptable is stem cell research for cerebral palsy?

◯ 0 - Totally unacceptable
◯ 1
◯ 2
◯ 3
◯ 4
◯ 5 - Neutral
◯ 6
◯ 7
◯ 8
◯ 9
◯ 10 - Perfectly acceptable

Survey 13% Complete

You will now be presented with some information on neural stem cells

There are many different types of stem cells. This survey is focusing on one type, neural stem cells, which can turn into the main cell types of the brain. Neural stem cells can reduce the damage caused by brain injury and therefore, can potentially reduce the severity of cerebral palsy.

There have been many animal studies conducted around the world showing that neural stem cells can be beneficial following brain injury. Additionally, neural stem cells have been tested in clinical trials as a potential treatmeant for disorders of the central nervous system such as stroke, Parkinson's disease and spinal cord injury. Therefore, neural stem cells could possibly be used to treat people diagnosed with cerebral palsy.

The following table below outlines examples of scientific outcomes following studies using infant animal models.

The benefit of neural stem cell therapy	Example of scientific evidence
**Reduces brain tissue loss**	Below are slices of brain tissue from mice. The green area shows a region of the brain and how it usually looks. The blue area shows how this area looks following brain injury.<br>Following neural stem cell treatment, there was a 50% reduction in the amount of brain tissue lost.<br>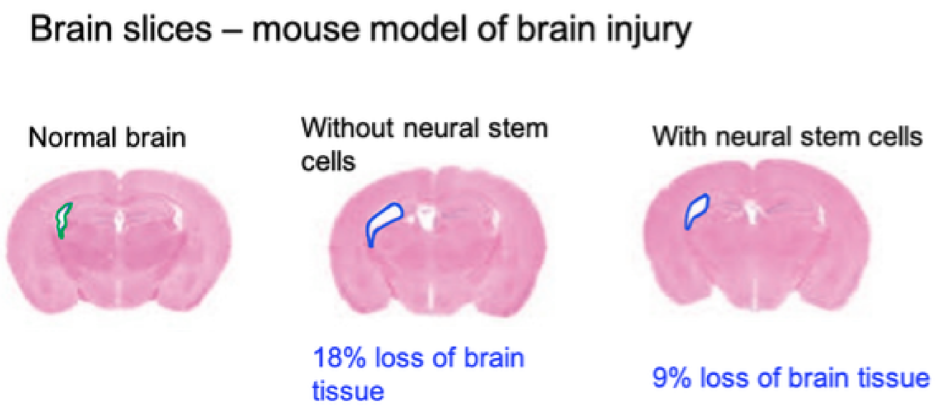
**Reduces brain inflammation**	The number of inflammatory cells, which can cause injury, in a specific region of the brain:<br>~ 8000 cells without neural stem cell therapy<br>~ 4000 cells with neural stem cell therapy<br>50% reduction in the number of inflammatory cells
**Improves motor deficits**	Improved ability of mice to grasp a bar when administered neural stem cells after brain injury<br><br>Improved ability of rats to balance and walk on a rotating beam when administered neural stem cells after brain injury

References: Braccioli et al 2017, Pediatric Research. 81, 127-135. Daadi et al 2010, Stroke. 41, 516-523. Lee et al 2017, Translational research. 183, 121-136

16) The next section will ask your opinion on neural stem cell therapy, please ensure that you have read and understood the above.
By clicking 'Continue', I acknowledge that I have:
Read and understood the above text

◯ Continue

Survey 20% Complete

4. How do we obtain neural stem cells?

Neural stem cells get their name ‘neural’ because they come from the brain. We, therefore, need to obtain these cells from brain tissue or produce brain cells from other cell types in the laboratory.

You will now be presented with information on three different sources of neural stem cells to understand whether you find each cell type acceptable.

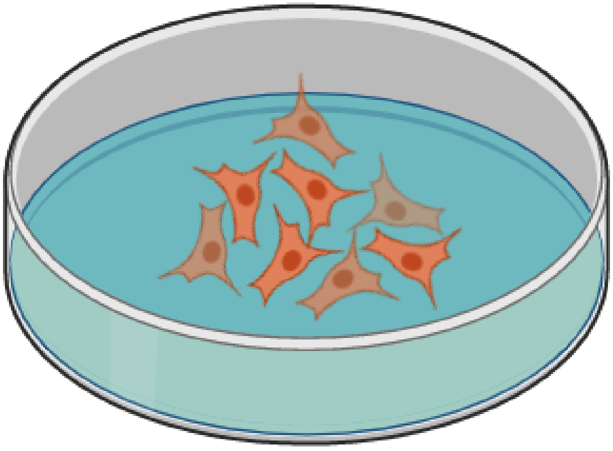

Neural stem cells can be sourced from fetal tissue. In this case, they are obtained from brain tissue of electively aborted fetuses with consent and as a donation to medical research. The neural stem cells are then isolated and grown in the laboratory, ready to use for research.

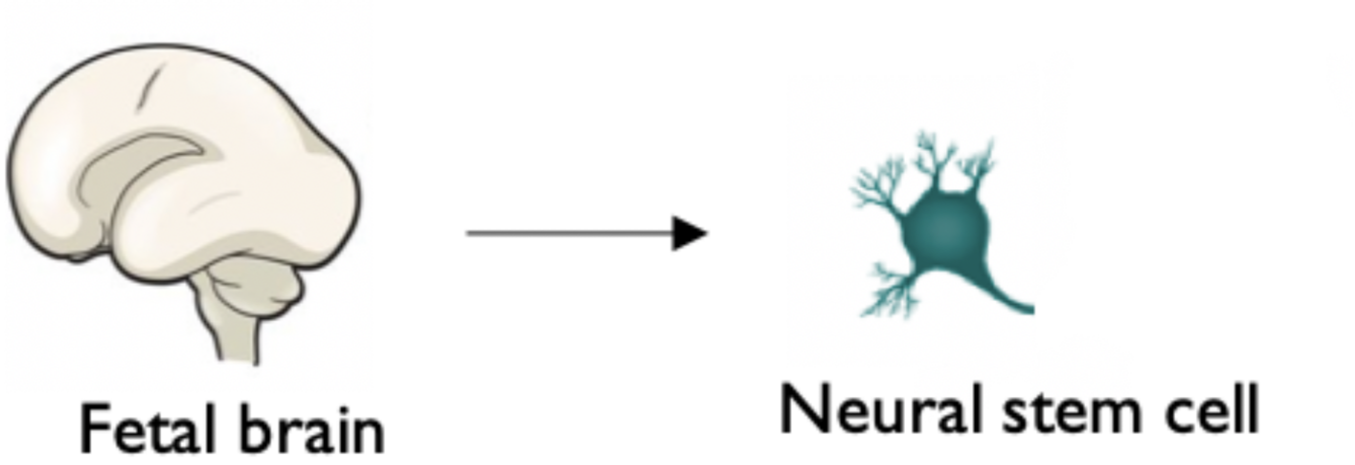

Would you be willing to use fetally-derived neural stem cells to treat cerebral palsy in yourself/your child?	◯ 0- Not willing to use<br>◯ 1<br>◯ 2<br>◯ 3<br>◯ 4<br>◯ 5 - Unsure<br>◯ 6<br>◯ 7<br>◯ 8<br>◯ 9<br>◯ 10 - Very willing to use
Do you have ethical concerns about using fetally-derived neural stem cells?	◯ Yes<br>◯ No
Please list your ethical concerns:	________________________

Survey 33% Complete

Furthermore, neural stem cells can be formed from embryos. They are sourced from embryos that are either unsuitable for IVF or are no longer required by the patient and are donated to medical research, with consent. Embryonic stem cells are obtained from the inner cell mass of the blastocyst (seen below). In normal development these cells would subsequently turn into all cells of the body, for example muscle and bone cells. When scientists extract these cells from the inner cell mass, they maintain their embryonic stem cell characteristics in the laboratory. These embryonic stem cells then require some laboratory manipulation to form neural stem cells.

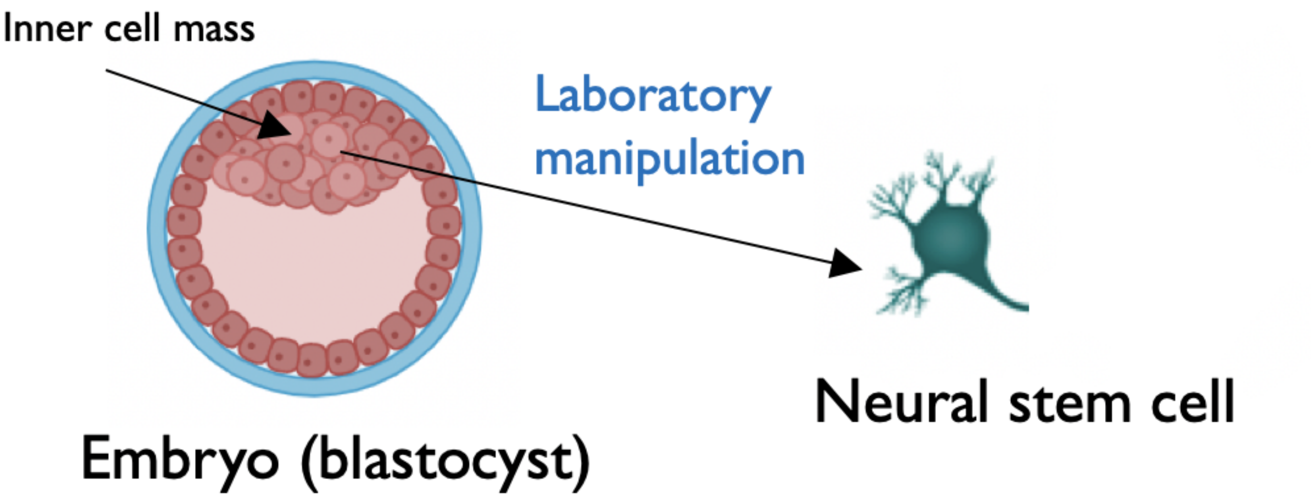

Would you be willing to use embryonically derived neural stem cells to treat cerebral palsy in yourself/your child?

- ◯ 0- Not willing to use
- ◯ 1
- ◯ 2
- ◯ 3
- ◯ 4
- ◯ 5 - Unsure
- ◯ 6
- ◯ 7
- ◯ 8
- ◯ 9
- ◯ 10 - Very willing to use

Do you ethical concerns about using embryonically-derived neural stem cells?

- ◯ Yes
- ◯ No

Please list your ethical concerns:

____

Survey 40% Complete

Adult cells can be engineered in the laboratory to form neural stem cells. These cells are not derived from the brain, they can be sourced from cells such as skin cells. These cells are called induced pluripotent stem (iPS) cells. An example of this process would be forming neural stem cells from adult skin cells (fibroblast cells).

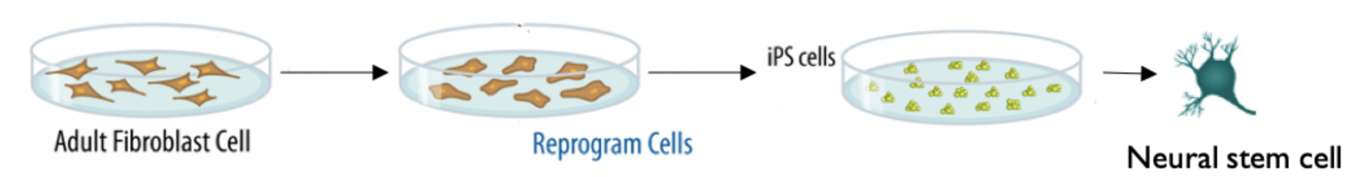

Would you be willing to use neural stem cells derived from induced pluripotent stem cells to treat cerebral palsy in yourself/your child?

◯ 0- Not willing to use
◯ 1
◯ 2
◯ 3
◯ 4
◯ 5 - Unsure
◯ 6
◯ 7
◯ 8
◯ 9
◯ 10 - Very willing to use

Do you have ethical concerns about using induced pluripotent derived neural stem cells?

◯ Yes
◯ No

Please list your concerns:

____

Survey 47% Complete

Availability of neural stem cells for research and clinical trials

Fetally-derived neural stem cells are ready to be used in human research trials, known as clinical trials. Some may consider these cells to be ethically controversial, since they are derived from fetal tissue.

In contrast, induced pluripotent stem cells are derived from adult cells, but are not yet available for human clinical trials. Induced pluripotent stem cell are not ready to use in humans because further research is required to determine their safety and effectiveness. This is summarised in the table below:

Neural stem cell	Source	Status
**Fetally-derived neural stem cells**	Fetal brain cells 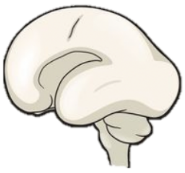	Ready to use in clinical trials
**Induced pluripotent derived neural stem cells**	Adult cells 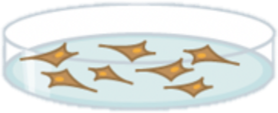	Needs more research into their safety

26) Would you be willing to use fetally-derived neural stem cells, or would you prefer to wait for an induced pluripotent stem cell to become available?

- ◯ Use fetally-derived stem cells
- ◯ Wait for induced pluripotent stem cells to become available

27) Before the next section commences, please provide any comments you have so far:

____

Survey 53% Complete

How do we give neural stem cells to patients?

Stem cells could be administered into the body to treat brain injury via:

- Intravenous infusion: stem cells delivered into the blood circulation via a drip

- Direct injection to the site injury: requires neurosurgery so stem cells can be directly injected to the site of injury, where they are needed

Scientists have considered both methods listed above to give neural stem cells. It is more likely that direct injection of neural stem cells into the brain would be the most effective way to deliver neural stem cells. This would involve neurosurgical injection of neural stem cells by a neurosurgeon under general anaesthetic (deep sleep during surgery).

Neural stem cells are different from other stem cell types, because to be most effective, they cannot be given via a drip. If a drip was used to deliver neural stem cells, they cannot travel effectively to the brain, which is were we want the cells to do their work in the brain and rebuild tissue.

You will now be presented with a table that 'weighs up' the benefits and risks/considerations of neurosurgical administration of neural stem cells.

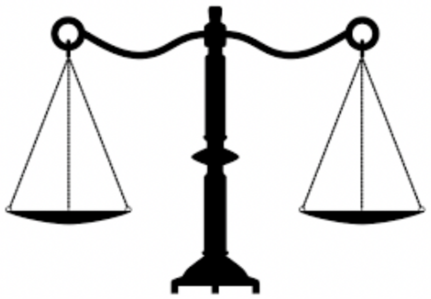

Neurosurgical injection of neural stem cells:

Benefits	Risks/considerations
• Can give neural stem cells directly to the site of brain injury<br>• Allows neural stem cells to be most effective<br>• The only method of giving neural stem cells which replaces dead brain cells	• 1-2% risk of bleeding within the brain as a result of neurosurgery<br>• 1-2% risk of infection as a result of neurosurgery<br>• Less than 1% risk of allergic reaction, breathing problems from anaesthetic

28) Knowing that neurosurgery is required to administer neural stem cells, how willing are you to use this therapy for yourself/your child?

- ◯ 0- Not willing to use
- ◯ 1
- ◯ 2
- ◯ 3
- ◯ 4
- ◯ 5 - Unsure
- ◯ 6
- ◯ 7
- ◯ 8
- ◯ 9
- ◯ 10 - Very willing to use

29) If you would like, please provide any comments:

Survey 60% Complete

Neural stem cells and the immune system

The immune system works to protect us from outside invaders by recognising foreign material, such as bacteria, that can make us sick. The immune system cannot distinguish between "bad" foreign material such as bacteria or "good" foreign material such as neural stem cells.

The immune system recognises neural stem cells as foreign because they come from a donor source, leading to neural stem cell rejection. This rejection is much like the potential rejection of a solid organ transplant such as the heart, lungs or kidneys.

This is a problem, because if neural stem cells are rejected by the immune system, they would be of no benefit. The longer neural stem cells can remain within the brain without being rejected, the more likely they are to repair brain damage.

Immunosupressive drugs can be used in conjunction with neural stem cell therapy to help prevent neural stem cell rejection. They work by suppressing the immune system to avoid rejection of a treatment like a neural stem cell.

Immunosuppressive drugs are likely to hold benefits and some risks when used in conjunction with neural stem cell therapy.

Below is a table that 'weighs up' the benefits and risks of immunosuppression:

You will now be presented with a table that ‘weighs up’ the benefits and risks/considerations of immunosuppression:

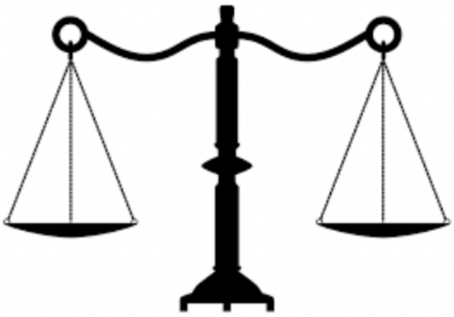

Immunosuppression in conjunction with neural stem cell therapy:

Benefits	Risks/considerations
• Prolonged neural stem cell survival in the brain → may allow for increased neural stem cell benefits<br>↓<br>May lead to greater motor/cognitive improvements	• Immunosuppression may be required for at least 9 months<br>• Reduced immune system function which increases the risk of getting sick

30) Knowing that immunosuppression is required in conjunction with neural stem cell therapy, how willing are you to use this therapy for cerebral palsy for yourself/your child?

- ◯ 0- Not willing to use
- ◯ 1
- ◯ 2
- ◯ 3
- ◯ 4
- ◯ 5 - Unsure
- ◯ 6
- ◯ 7
- ◯ 8
- ◯ 9
- ◯ 10 - Very willing to use

31) If you would like, please provide any additional comments:

Survey 67% Complete

Early evidence in neural stem cell therapy suggests that the earlier the stem cell administration, the better the outcome. Are you in favour of using neural stem cells, with neurosurgical intervention and an immunosuppressive regime, in:

32) Newborn, 0-3 months old:

- ◯ 0- Strongly oppose
- ◯ 1
- ◯ 2
- ◯ 3
- ◯ 4
- ◯ 5 - Unsure
- ◯ 6
- ◯ 7
- ◯ 8
- ◯ 9
- ◯ 10 - Strongly support

33) Infant, 3-18 months:

- ◯ 0- Strongly oppose
- ◯ 1
- ◯ 2
- ◯ 3
- ◯ 4
- ◯ 5 - Unsure
- ◯ 6
- ◯ 7
- ◯ 8
- ◯ 9
- ◯ 10 - Strongly support

34) Child, 18 months and over:

- ◯ 0- Strongly oppose
- ◯ 1
- ◯ 2
- ◯ 3
- ◯ 4
- ◯ 5 - Unsure
- ◯ 6
- ◯ 7
- ◯ 8
- ◯ 9
- ◯ 10 - Strongly support

35) Teenager:

- ◯ 0- Strongly oppose
- ◯ 1
- ◯ 2
- ◯ 3
- ◯ 4
- ◯ 5 - Unsure
- ◯ 6
- ◯ 7
- ◯ 8
- ◯ 9
- ◯ 10 - Strongly support

36) Adult:

- ◯ 0- Strongly oppose
- ◯ 1
- ◯ 2
- ◯ 3
- ◯ 4
- ◯ 5 - Unsure
- ◯ 6
- ◯ 7
- ◯ 8
- ◯ 9
- ◯ 10 - Strongly support

Survey 73% Complete

Future research into neural stem cells

Clinical trials are research investigations in which people volunteer to test new treatments for medical conditions. Clinical trials ensure that a new intervention is safe and effective.

In the future, clinical trials will need to be conducted to test the safety and effectiveness of all aspects of neural stem cell therapy, including neurosurgerical delivery of neural stem cells and immunosuppressive drug regimes.

---

37) What is the likelihood you would elect to have you/your child undergo neural stem cell therapy for cerebral palsy in a clinical trial?

- ◯ 0- Extremely unlikely
- ◯ 1
- ◯ 2
- ◯ 3
- ◯ 4
- ◯ 5 - Unsure
- ◯ 6
- ◯ 7
- ◯ 8
- ◯ 9
- ◯ 10 - Extremely likely

---

38) Once a clinical trial is complete and if neural stem cells have been shown to be safe and effective, what is the likelihood you would elect to undergo neural stem cell therapy?

- ◯ 0- Extremely unlikely
- ◯ 1
- ◯ 2
- ◯ 3
- ◯ 4
- ◯ 5 - Unsure
- ◯ 6
- ◯ 7
- ◯ 8
- ◯ 9
- ◯ 10 - Extremely likely

---

39) If you would like, please provide any additional comments:

Survey 80% Complete

This question will ask you to consider a hypothetical scenario about neural stem cell therapy

An infant (less than 6 months old) has been diagnosed with cerebral palsy and is a candidate to receive neural stem cells neurosurgically. The likely considerations are:

Risks Benefit

- 15% increased risk of infection (such as chest infection, pneumonia requiring hospitalisation) following neurosurgery and immunosuppression

- 2% risk of other adverse events (such as bleeding following neurosurgery)

- 10-20% improvement in motor function

---

40) If this was your child and given the above considerations, would you be willing to undertake neural stem cell therapy to treat cerebral palsy?

◯ 0- Not willing
◯ 1
◯ 2
◯ 3
◯ 4
◯ 5 - Unsure
◯ 6
◯ 7
◯ 8
◯ 9
◯ 10 - Very willing

---

41) Based on you own personal experience with cerebral palsy - If you were to consider a stem cell treatment in the future, what would a small but significant improvement in motor function look like to you/your child following this treatment?

Survey 87% Complete

Stem cell therapy more broadly

This survey has provided detailed descriptions of neural stem cell therapy.

Once again, we would like you to consider stem cell therapies more broadly.

42) How acceptable is stem cell research?

◯ 0 - Totally unacceptable
◯ 1
◯ 2
◯ 3
◯ 4
◯ 5 - Neutral
◯ 6
◯ 7
◯ 8
◯ 9
◯ 10 - Perfectly acceptable

43) How acceptable is stem cell research for cerebral palsy?

◯ 0 - Totally unacceptable
◯ 1
◯ 2
◯ 3
◯ 4
◯ 5 - Neutral
◯ 6
◯ 7
◯ 8
◯ 9
◯ 10 - Perfectly acceptable

Survey 93% Complete

Thank you for your input so far!

44) Here is some space to provide final comments about any aspect of this survey:

45) Do you consent to researchers contacting you in the future about this research and other stem cell related research?
This will include providing a report of results from this survey once this study is completed.

- ◯ Yes
- ◯ No

Disclaimer

We have discussed the potential benefits of neural stem cell therapy in great detail, but please note that neural stem cell therapy is not a proven treatment for cerebral palsy and other neurological disorders. They have not received regulatory approval for use in Australia, the US and Canada.

We have discussed positive results from animal studies, but these results do not always translate to humans. To determine if these results translate to humans, clinical trials are required before this therapy may be deemed safe and effective. We encourage engagement with research, but it is important to make sure you are engaging with reputable organisations.

You should always discuss medical decisions for your/your child’s treatment with your medical doctor.

If you would like more information please refer to the links below:

http://www.stemcellsaustralia.edu.au/About-Stem-Cells.aspx

https://www.closerlookatstemcells.org/learn-about-stem-cells/stem-cell-basics/

If this survey caused any distress and you wish to speak to someone please call Lifeline on 13 11 14 (Australia), National Suicide Prevention Lifeline on 1800 273 8255 (United States) or Canada Suicide Prevention Service on 1 833 456 4566 (Canada).

If you have any further questions please contact Madeleine Smith: madeleine.smith@monash.edu

If you have any concerns and/or complaints about this project, the way it has been conducted or your rights as research participants, and would like to speak with someone independent of the project, please contact The University of Sydney Human Research Ethics Committee Tel: +61 2 8627 0200

Please feel free to share the link to this survey with someone you think may be eligible and interested in participating

To share, copy and paste the link below:

https://www.sydney.edu.au/research/volunteer-for-research-study/other/stem-cell-therapy-cerebral-palsy.html

Thank you for taking the survey. Your contribution to neural stem cell research is greatly appreciated.

Have a nice day!

Survey 100% Complete
